# Comprehensive evaluation of gait, spasticity, and muscle morphology: A case report of a child with spastic paresis treated with Botulinum NeuroToxin‐A, serial casting, and physiotherapy

**DOI:** 10.1002/ccr3.2227

**Published:** 2019-07-21

**Authors:** Guido Weide, Lizeth Sloot, Laura Oudenhoven, Richard T. Jaspers, Jaap Harlaar, Annemieke I. Buizer, Lynn Bar‐On

**Affiliations:** ^1^ Laboratory for Myology, Department of Human Movement Sciences, Amsterdam Movement Sciences Vrije Universiteit Amsterdam Amsterdam The Netherlands; ^2^ Department of Rehabilitation Medicine, Amsterdam Movement Sciences Amsterdam UMC, location VUmc Amsterdam The Netherlands; ^3^ Department of Biomechanical Engineering Delft University of Technology Delft The Netherlands; ^4^ Department of Rehabilitation Sciences KU Leuven Leuven Belgium

**Keywords:** Botulinum toxin, cerebral palsy, foot flexibility, gait, gastrocnemius medialis muscle, intervention, muscle morphology, spasticity, ultrasound

## Abstract

Comprehensive instrumented muscle and joint assessments should be considered when prescribing Botulinum NeuroToxin‐A (BoNT‐A) treatment in spastic paresis. In a child with spastic paresis, comprehensive evaluation following treatment with BoNT‐A, serial casting, and physiotherapy showed that short‐term improvements in gait occurred without changes in muscle morphology. Rather, foot flexibility increased.

## 1 INTRODUCTION

In children with spastic paresis (SP), gait deviations, including limited dorsiflexion during the stance phase, are generally attributed to calf muscle spasticity and non‐neural changes in soft tissue properties.^1^ Therefore, to improve gait in children with SP, the medial gastrocnemius is frequently treated with intramuscular BoNT‐A injections. BoNT‐A results in a temporary blockage of the neurotransmission of acetylcholine to the nerves motor endplates.^2^ To target the changes in soft tissue properties that contribute to reduced ankle dorsiflexion, BoNT‐A is often combined with serial casting of the lower leg such that the plantar flexor muscles are gradually stretched. In such a combined treatment approach, it is presumed that after decreasing muscular activation with BoNT‐A, serial casting of muscles at an extended length stimulates the addition of sarcomeres in series, increases their lengths, and reduces their stiffness.^3^ Currently, human models that substantiate these working mechanisms do not exist.^4^, ^5^

Increases in ankle joint range of motion (ROM) in SP after BoNT‐A and serial casting have been reported.^6^, ^7^, ^8^ In routine clinical assessment, this ankle joint ROM is determined by examining the angle between the foot sole and the shank. However, using the orientation of the foot can be erroneous since the ankle joint and foot comprise multiple articulating bones. This is especially important since clinicians often use this examination of “foot” ROM to infer about triceps surae muscle extensibility.^9^ In addition, routine clinical assessments cannot adequately quantify the contribution of spasticity and changes in soft tissue properties to reduced ankle ROM.^10^ Therefore, more comprehensive, instrumented evaluations^11^, ^12^, ^13^ that provide better insight into the working mechanisms of treatment with BoNT‐A, are required in clinical practice. These can improve treatment rationale and may prevent the use of ineffective or even harmful treatments.

Here, we present a case study of a 6‐year‐old girl with SP who was treated with BoNT‐A injections in her calf muscles, serial lower leg casting, and physiotherapy. The aim of the study was to evaluate the effects of this treatment on gait and relate changes in the gait to changes occurring at the joint and muscle level. To do this, we carried out a comprehensive instrumented assessment of the ankle joint and plantar flexor muscle morphology and spasticity before, at 9 and 26 weeks post‐treatment.

## 2 CASE HISTORY

A 6‐year‐old girl (120 cm tall, 21 kg) diagnosed with bilateral SP due to unknown etiology and greater involvement of her right side participated in this study. The pregnancy and birth history of the patient were unremarkable. Her brain MRI showed no abnormalities, thereby excluding the diagnosis of cerebral palsy. Genetic testing was done because hereditary spastic paraplegia (HSP) was suspected. The patient had no family history of SP. No mutations were found in genes associated with HSP (SPG4, SPG7, REEP1, and ATL1). Whole exome sequencing did not offer a diagnosis. Metabolic testing revealed no abnormities, excluding a metabolic cause of the SP. Therefore, it was concluded that there was SP of unknown origin. Most likely, the genetic cause is not yet known. Renewed genetic testing is planned in five years’ time in case new genes have been found to be associated with SP.

She was diagnosed with developmental dysplasia of the right hip for which she wore a hip abduction brace from 9 to 11 months of age. She was able to walk without aids from the age of 17 months. At the age of three, she was prescribed bilateral ankle‐foot orthoses (AFOs) that she wore during the day. She also received physiotherapy 1‐2 times/wk aimed to improve her walking‐related activity goals.

With age, she developed specific gait deviations including increased knee flexion and reduced ankle dorsiflexion at initial contact, midstance, and swing. Physical examination and 2D video gait analysis were employed to identify the underlying impairments.^14^ Clinical examination revealed spasticity in the calf muscles and reduced ankle ROM. To reduce spasticity, increase ankle ROM, and improve gait, three sessions of multilevel BoNT‐A interventions combined with serial casting were prescribed when she was 3, 4, and 5 years old. The aim of the serial casting was to increase m. triceps surae length while BoNT‐A injections would reduce spasticity and thereby facilitate muscle lengthening.

The current study was initiated when it was decided to use serial casting and BoNT‐A injections for the fourth time. At this time, her parents reported that she had pain when wearing the AFOs and was therefore unable to walk long distances. Physical examination^14^ revealed a passive ankle dorsiflexion ROM of −25° with the knee extended in the right leg and −20° in the left leg. Spasticity was clinically diagnosed by the perception of a catch during fast passive stretch^14^ bilaterally in the gastrocnemius, soleus, hamstrings, and adductor muscles. Her gait pattern was characterized by forefoot contact on landing with excessive knee and hip flexion in midstance (Type 4 pattern^14^). The goal of the intervention was to improve the duration of wearing the AFOs by improving ankle ROM. BoNT‐A injections were administered under general anesthesia (Table 1). Three weeks after injection, serial casting was applied on both legs from below the knee. The patient was instructed to stand and walk regularly with the casts. Weekly, the casts were changed to allow incremental correction of the foot form and of ankle angle. Physiotherapy was intensified (30‐45 minutes 3× per wk) and continued up to 12 weeks after the injections while the use of the AFOs was continued after the casts were removed. Post‐treatment physiotherapy goals included correction of foot and knee positioning during gait, and improving walking distance.

**Table 1:** Muscles that received BoNT‐A injections of Botox^®^

	Right (units)	Left (units)
m. psoas	40	40
Adductors	2 × 20	—
m. gracilis	2 × 20	2 × 20
m. semimembranosus	2 × 20	2 × 20
m. semitendinosus	2 × 20	2 × 20
m. gastrocnemius medialis	2 × 20	—
m. soleus	2 × 15	—
	Total units: 360	

**Figure 1:**
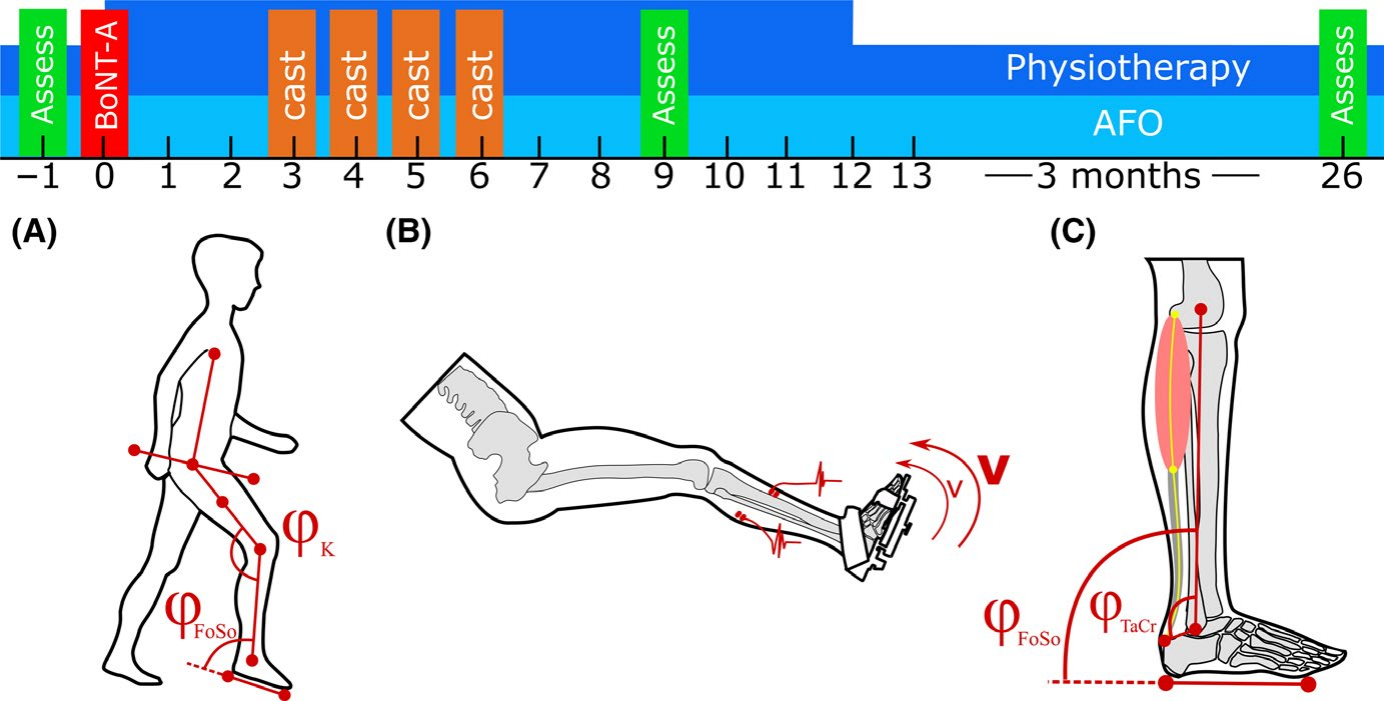
Timeline of the assessments and treatments (BoNT‐A and casting) alternated with periods of conventional physiotherapy and bilateral ankle‐foot orthoses (AFO). Assessments were carried out −1 wk pre, 9 wk and 26 wk post‐treatment. Assessments: (A) 2D gait analysis, assessing walking velocity, stride time, stride length and foot sole (*φ*_FoSo_), and knee angles (*φ*_K_) at initial contact and at midstance; (B) instrumented spasticity assessment of the m. gastrocnemius medialis and the m. tibialis anterior performed at slow (*v*) and fast (*V*) angular velocities; and (C) foot sole range of motion, foot flexibility, and 3D ultrasound of m. gastrocnemius medialis morphology (including estimated talocrural joint angle (*φ*_TaCr_), muscle‐tendon complex, muscle belly, and tendon lengths) at foot sole angles (*φ*_FoSo_) corresponding to standardized externally applied dorsiflexion footplate moments (i.e., 0 and 4 Nm)

In addition to the routine clinical examination and 2D video gait, the following assessments were carried out 1 week before the BoNT‐A injections (−1 wk) and 9 weeks (9 wk) as well as 26 weeks (26 wk) post‐treatment: (a) instrumented spasticity assessment of the calf muscles,^11^ (b) foot ROM and foot flexibility measurements,^15^ and (c) muscle morphometry through 3D ultrasound imaging^12^ (Figure 1). The clinical examination and 2D video gait analysis were carried out by the physician (AB) and hospitals’ laboratory technician (LO) while the additional instrumented measurements were carried out by researchers (GW, LS) trained in human movement sciences and by a postdoctoral researcher trained in physiotherapy and biomedical sciences (LB).

Ethical approval for the study design was granted from the Amsterdam UMC medical ethical committee. Both the patients’ parents provided informed consent.

## 3 INVESTIGATIONS

### 3.1 Gait analysis (GA)

The patient underwent routine barefoot clinical GA that involved walking over a 10‐m walkway at self‐selected walking speed. Video recordings were taken in the sagittal plane. MoXie Viewer^®^ software was used to measure sagittal knee and ankle angles at initial contact and midstance over six representative strides (Figure 1A).^16^ The joint angles were determined as follows: knee angle (*φ*_K_) was defined as the angle between two lines representing the shank and thigh, and foot sole angle (*φ*_FoSo_) was defined as the angle between two lines representing the shank and foot. Spatio‐temporal parameters, including walking velocity, normalized walking velocity,^17^ stride time, and relative stance phase time, were calculated based on leg length and the timing of foot strike and foot‐of.

### 3.2 Instrumented spasticity assessment

Instrumented spasticity assessment was carried out using a motor‐driven footplate (MOOG, Nieuw‐Vennep, The Netherlands).^11^, ^18^ The patient was seated in an adjustable chair with the right foot fixed onto a custom designed adjustable footplate^15^, ^19^ (Figure 1C). This footplate allowed adjustments targeted to fix the talocalcaneal joint during foot plate rotations (for details see Huijing et al.^15^) The motor‐driven footplate applied two slow (15°/s) and two fast (150°/s) speed controlled dorsiflexion movements over the patient's maximum ankle ROM (determined manually). Muscle excitation during rotations was measured using surface electromyography (EMG) from the m. tibialis anterior (TA) and GM. Preparation of the skin and placements of the EMG electrodes were performed according to SENIAM guidelines and confirmed with ultrasound imaging.^20^ Data from the TA were used to exclude for the possibility of voluntary activation aiding dorsiflexion. The minimum baseline (averaged from −0.5 to 0 second before movement) RMS‐EMG was subtracted from the maximum (calculated as the 95th percentile to correct for outliers). This corrected maximum RMS‐EMG was averaged for slow and fast stretches separately. To quantify spasticity (velocity‐dependent stretch reflexes) in GM, the average RMS‐EMG value during slow stretches was subtracted from the average value during fast stretches.

### 3.3 Joint angles and foot flexibility

The patient was positioned prone on an examination table with both feet overhanging the edge of the table. An identical footplate as used in the instrumented spasticity assessment was fitted to the patient's foot. Subsequently, an inclino‐dynamometer was connected to the footplate. Next, foot sole angles (*φ*_FoSo_), defined with respect to the shank were determined corresponding to 0 and 4 Nm externally applied dorsiflexion moments. At each *φ*_FoSo_, talocrural joint angle (*φ*_TaCr_), defined as the angle between the insertion of the Achilles tendon onto the calcaneus, the central point between the malleoli and the central point between the femoral epicondyles, was determined by retrieving the coordinates of bony landmarks using a 3D stylus (Figure 1C). Positive angles correspond to dorsiflexion angles. The difference between *φ*_TaCr_ and *φ*_FoSo_ angles (*φ*_TaCr_−*φ*_FoSo_) represents the difference in orientation of the hindfoot with respect to the foot sole (Figure 1C). Decreases in *φ*_TaCr_−*φ*_FoSo_ with increases in externally applied dorsiflexion moment between 0 and 4 Nm are considered as estimates of the effect of foot flexibility.^21^

### 3.4 Muscle morphology

At *φ*_FoSo_ corresponding to 0 and 4 Nm, B‐mode 3D ultrasound (3DUS) images were collected.^12^ From those, morphological characteristics, including muscle‐tendon complex length ($\ell_{\text{m + t}}$), muscle belly length ($\ell_{\text{m}}$), and tendon length ($\ell_{\text{t}}$), were measured.^12^ Length changes between 0 and 4 Nm were calculated and normalized to lower leg length ($\ell_{\text{ll}}$). Length changes of $\ell_{\text{m}}$ and $\ell_{\text{t}}$ were also expressed as percentages of $\ell_{\text{m + t}}$ length changes. Finally, at *φ*_FoSo_ corresponding to 0 Nm, muscle volume (*V*_GM_), fascicle length ($\ell_{\text{fasc}}$), and physiological cross‐sectional area (*A*_fasc_) were determined.^12^ *V*_GM_ and *A*_fasc_ were normalized for body mass (BM).

## 4 OUTCOME AND FOLLOW‐UP

The results of the patient's routine 2D video gait analysis are reported below, followed by results from the additional instrumented assessments (Table 2).

**Table 2:** Overview of the mean outcome values of gait analysis (Gait), instrumented spasticity assessment (Spast), ankle range of motion (ROM), and gastrocnemius medialis muscle morphology 3D ultrasound (3DUS) assessments

Parameters			−1 wk	9 wk	26 wk
Gait		Walking velocity (m/s)	0.84	1.02	1.01
		Normalized walking velocity	0.36	0.42	0.41
		Mean stride time (s)	0.94	0.84	0.82
		Stride length (m)	0.79	0.85	0.83
	*φ*_FoSo_ (°)	Initial contact	−10.17	−7.83	−9.67
		Midstance	−4.33	5.67	0.67
	*φ*_K_ (°)	Initial contact	32.17	34.83	42.83
		Midstance	15.00	12.17	30.33
Spasticity	EMG (μV)	Max GM slow	10.3	23.2	17.3
		Max GM fast	66.5	25.6	37.8
ROM	*φ*_FoSo_ (°)	0 Nm	−30.1	−17.0	−22.8
		∆0‐4 Nm	18.4	22.2	18.9
	*φ*_TaCr_ (°)	0 Nm	24.4	29.1	17.0
		∆0‐4 Nm	13.1	11.5	15.0
	*φ*_TaCr_−*φ*_FoSo_ (°)	0 Nm	54.5	46.0	39.9
		∆0‐4 Nm	5.2	10.8	4.0
3DUS	*V*_gm_/BM (ml/kg)	0 Nm	1.6	1.6	1.7
	$\ell_{\text{fasc}}/\ell_{\text{ll}}$ (%)	0 Nm	14.9	14.7	14.2
	*A*_fasc_/BM (mm^2^/kg)	0 Nm	39.7	40.7	43.7
	$\ell_{\text{m + t}}/\ell_{\text{ll}}$ (%)	0 Nm	100.8	102.5	100.9
		∆0‐4 Nm	7.4	5.0	7.3
	$\ell_{\text{m}}/\ell_{\text{ll}}$ (%)	0 Nm	52.2	53.3	51.4
		∆0‐4 Nm	4.4	2.6	4.2
	$\ell_{\text{t}}/\ell_{\text{ll}}$ (%)	0 Nm	48.6	49.3	49.5
		∆0‐4 Nm	2.9	2.4	3.1
	$\Delta \ell_{\text{m}}$ (0‐4 Nm)/$\Delta \ell_{\text{m + t}}$ (0‐4 Nm) (%)		59.5	52.0	57.3
	$\Delta \ell_{\text{t}}$ (0‐4 Nm)/$\Delta \ell_{\text{m + t}}$ (0‐4 Nm) (%)		39.2	48.0	42.5

Notes

*φ*_FoSo_ = foot sole angle relative to lower leg; *φ*_K_ = thigh angle relative to lower leg; *φ*_TaCr_ = angle between the line connecting the insertion of the GM at the calcaneus with the center of the bimalleolar axis and the line following the lower leg; *φ*_TaCr_−*φ*_FoSo_ = the angle of the line connecting the insertion of the GM at the calcaneus with the center of the bimalleolar axis relative to the foot sole; positive angles indicate dorsiflexion angles, if delta angular values are reported positive, this indicates angular changes in dorsiflexion direction. *V*_gm_/BM = m. gastrocnemius medialis volume normalized for body mass; *A*_fasc_/BM (mm^2^/kg) = physiological cross‐sectional area normalized for body mass; $\ell_{\text{m + t}}/\ell_{\text{ll}}$ = muscle‐tendon complex length normalized for lower leg length; $\ell_{\text{m}}/\ell_{\text{ll}}$ = muscle belly length normalized for lower leg length; $\ell_{\text{t}}/\ell_{\text{ll}}$ = tendon length normalized for lower leg length; $\Delta \ell_{\text{m}}$ (0‐4 Nm)/$\Delta \ell_{\text{m + t}}$ (0‐4 Nm) = muscle belly lengthening between 0 and 4 Nm relative to muscle‐tendon complex lengthening between 0 and 4 Nm; $\Delta \ell_{\text{t}}$ (0‐4 Nm)/$\Delta \ell_{\text{m + t}}$ (0‐4 Nm) = tendon lengthening between 0 and 4 Nm relative to muscle‐tendon complex lengthening between 0 and 4 Nm.

### 4.1 Gait analysis

At 9‐wk follow‐up, absolute and normalized walking velocity had slightly improved (i.e., absolute velocity increase of 0.2 m/s) by a decreased stride time (−10.6%) and an increased stride length (7.6%) compared with pre‐treatment. In addition, kinematics showed that *φ*_FoSo_ at both initial contact and midstance had increased toward dorsiflexion by 2.3° and 10.0°, respectively. Although there was 2.7° more knee flexion at initial contact, there was 2.8° less knee flexion during midstance.

Similar to changes measured after 9 wk, after 26 wk, both normalized and absolute walking velocity increased with respect to pre‐intervention velocity (i.e., absolute velocity increase of 0.2 m/s), as stride time was lower (−14.6%) and stride length was longer (4.8%). However, gait kinematics showed that *φ*_FoSo_ at initial contact and midstance had almost returned to −1 wk values, (i.e., 0.5° and 5° more dorsiflexion, respectively). At 26 wk, knee extension had deteriorated compared to −1 wk, as both initial contact and midstance as *φ*_K_ flexion increased by 10.7° and 15.3°, respectively. The results of the gait analyses therefore indicate that only short‐term improvements were achieved.

### 4.2 Instrumented spasticity assessment

RMS‐EMG of the GM during fast dorsiflexion movements was considerably higher compared to that during slow movements, indicating the presence of velocity‐dependent involuntary muscular hyperactivity. After treatment, the velocity‐dependent hyperactivity (i.e., difference in activation of GM between slow and fast dorsiflexion movements) decreased by −52% at 9 wk and by −20% at 26 wk (Table 2).

### 4.3 Joint angles and foot flexibility

After 9 wk, *φ*_FoSo_ at 0 Nm had increased by 13.1° dorsiflexion. In addition, *φ*_FoSo_ range between 0 and 4 Nm had increased by 3.8°. After 26 wk, *φ*_FoSo_ at 0 Nm was still increased (by 7.3° dorsiflexion), but *φ*_FoSo_ range between 0 and 4 Nm had returned to almost pre‐intervention values (just a 0.5° increase).

After 9 wk, *φ*_TaCr_ at 0 Nm had increased toward dorsiflexion by 4.7°. However, after 26 wk, *φ*_TaCr_ had decreased toward plantarflexion by 7.4°. The *φ*_TaCr_ range between 0 and 4 Nm was decreased by 1.6° after 9 wk and was increased by 3.5° after 26 wk.

During follow‐up, *φ*_TaCr_−*φ*_FoSo_ at rest (i.e., at 0 Nm) was decreased (by 8.5° at 9 wk and by 14.6° at 26 wk), which suggests that bones in the foot at rest had changed their orientation with respect to the footplate. Decreases in *φ*_TaCr_−*φ*_FoSo_ at 0 Nm likely indicate that post‐treatment, the hind foot was more parallel with the foot sole. Change in *φ*_TaCr_−*φ*_FoSo_ between 0 and 4 Nm had increased by 5.6° at 9 wk and decreased by −1.2° at 26 wk compared with *φ*_TaCr_−*φ*_FoSo_ between 0 and 4 Nm at −1 wk. After 9 wk, flexibility of the foot had contributed to almost half of the *φ*_TaCr_−*φ*_FoSo_ between 0 and 4 Nm. After 26 wk, the effects of foot flexibility had reduced with respect to the −1 wk assessment. Though at 26 wk there was less flexion within the foot between 0 and 4 Nm, the foot was more deformed at 0 Nm, with a larger plantarflexion *φ*_TaCr_ with respect to *φ*_FoSo_.

### 4.4 Muscle morphology

During follow‐up, normalized muscle volume did not change. After 9 wk, normalized muscle‐tendon complex length ($\ell_{\text{m + t}}/\ell_{\text{ll}}$) at 0 Nm had increased by 1.7%. However, length changes of $\ell_{\text{m + t}}/\ell_{\text{ll}}$ between 0 and 4 Nm had decreased by −2.4% compared with those before the intervention, indicating that while $\ell_{\text{m + t}}/\ell_{\text{ll}}$ got longer, extensibility of the GM had decreased. After 26 wk, $\ell_{\text{m + t}}/\ell_{\text{ll}}$ at 0 Nm and GM extensibility between 0 and 4 Nm had returned to pre‐intervention levels.

After 9 wk, both muscle belly ($\ell_{\text{m}}/\ell_{\text{ll}}$) and tendon length ($\ell_{\text{t}}/\ell_{\text{ll}}$) corresponding to 0 Nm had increased by 1.1% and 0.7%, respectively. However, extensibility between 0 and 4 Nm decreased by −1.8% for $\ell_{\text{m}}/\ell_{\text{ll}}$ and by −0.5% for $\ell_{\text{t}}/\ell_{\text{ll}}$. After 26 wk, at 0 Nm, $\ell_{\text{m}}/\ell_{\text{ll}}$ had decreased by −0.8% and $\ell_{\text{t}}/\ell_{\text{ll}}$ had increased by 0.9%. Lengthening of $\ell_{\text{m}}/\ell_{\text{ll}}$ between 0 and 4 Nm had decreased by −0.2% and had increased for $\ell_{\text{t}}/\ell_{\text{ll}}$ by 0.2%. These findings indicate that, while $\ell_{\text{m}}/\ell_{\text{ll}}$ got shorter and $\ell_{\text{t}}/\ell_{\text{ll}}$ got longer after 26 wk, muscle belly lengthening and tendon lengthening relative to the muscle‐tendon complex lengthening decreased. With shorter $\ell_{\text{m}}/\ell_{\text{ll}}$ at 9 and 26 wk, normalized fascicle length ($\ell_{\text{fasc}}/\ell_{\text{ll}}$) had slightly decreased following treatment. While normalized GM volume did not change, physiological cross‐sectional area normalized for body mass (*A*_fasc_/BM) slightly increased following treatment.

## 5 DISCUSSION

In this case study, we found short‐term improvements in gait that were accompanied by a large reduction in calf muscle hyperactivity and improved ankle ROM after BoNT‐A injections combined with serial casting and physiotherapy in a child with SP. However, increased flexibility of the foot, rather than changes in GM morphology largely explained the increased ankle ROM. These results suggest that improvements in gait were predominantly due to reduction in muscle hyperactivity and increased foot flexibility, and not to change in muscle morphology.

### 5.1 Short‐term effects on gait

Gait characteristics improved with regard to ankle (foot sole) dorsiflexion angles as expected based on previous studies.^3^, ^22^, ^23^, ^24^ Improvements in gait following denervation by local BoNT‐A injections and serial casting may be due to the following: (a) temporary denervation causing a reduction in muscle hyperactivity,^25^ (b) changes in plantar flexor muscle morphology and/or stiffness,^26^, ^27^ and (c) increased tolerance to stretch.

At 9 wk, passive ankle ROM had improved and velocity‐dependent stretch reflexes were reduced. Similar to our findings, other studies have also reported short‐term increased ankle (foot sole) ROM as a result of BoNT‐A treatment.^28^ However, we found no changes in muscle morphology, indicating that changes in passive ankle ROM during gait were not because of morphological changes in the GM. This finding is also supported by recent studies of Pothrat et al. and Kalkman et al. in which changes in *φ*_FoSo_ did not correspond to length changes of the triceps surae muscles.^29^, ^30^

Instead, it is likely that increased foot flexibility contributed to observed changes in gait. Using a simple approach, we showed that flexibility of the foot greatly contributed to apparent ankle ROM, especially at 9 wk post‐treatment. During passive ankle ROM assessment with a maximally externally applied 4 Nm dorsiflexion footplate moment, almost half of the *φ*_FoSo_ ROM was accounted for by flexion within the foot. Therefore, it is expected that during gait, when much higher loads are imposed, foot flexibility will explain a substantial fraction of *φ*_FoSo_ change.^29^ Altogether, our findings question whether the treatment goal of increasing ankle ROM in this case was achieved. In addition, it challenges other positive findings of increased ankle ROM reported in literature.

### 5.2 Long‐term effects on gait

Half a year after the intervention, overall gait had deteriorated with respect to values measured pre‐intervention. Knee flexion angles during midstance substantially increased (i.e., deteriorated) and ankle angles during gait returned to pre‐intervention values. Based on previous studies showing that functional improvements in children with cerebral palsy after BoNT‐A could last up to 6 months, it was expected that the effects of BoNT‐A injections would only yield temporarily.^31^ In line with the observed return in limitations in ankle dorsiflexion during gait, we also observed a slight increase in muscle hyperactivity. This was also expected as in mice, stretch reflexes recovered 28 days after injection.^32^ In addition, at 26 wk, the passive ankle ROM value was worse than at 9 wk post‐treatment. The combination of both a return in muscle hyperactivity and a decrease in passive ankle ROM suggests that the intended effects of treatment had disappeared. Compared to short‐term, less flexibility within the foot occurred at 26 wk, indicating that changes in *φ*_FoSo_ ROM were now presumably more associated with triceps surae extensibility. Tissue stiffness at 26 wk had also returned to pre‐treatment values as indicated by muscle and tendon extensibility between 0 and 4 Nm ankle moments. In addition, at 26 wk an unwanted increase in mid‐stance knee flexion angle in gait was found, which may be related to a recurrence of hyperactivity of the GM. Therefore, on the long term, we found no benefit of the treatment with even a deterioration of knee angles during gait.

### 5.3 Marginal treatment effects on muscle morphology

The physiological cross‐sectional area (*A*_fasc_) of the GM marginally increased after treatment. It is generally presumed that BoNT‐A injections combined with serial casting improve the extensibility of triceps surae muscles by reducing muscular hyperactivity, by atrophy, and by length adaptations of muscle fibers.^33^, ^34^, ^35^ Muscle atrophy implies a reduction in the number of titin filaments arranged in parallel,^36^ which is associated with reduced resistance to extension.^37^ A reduction in stretch resistance caused by denervation or by atrophy might allow the muscle belly to stretch to extended lengths during serial casting. This is expected to induce an addition of sarcomeres in series, which will shift the optimum muscle length toward a longer length (i.e., dorsiflexion). However, our results show that following BoNT‐A treatment neither atrophy nor substantial adaptation in GM length were accomplished. Muscle strains could explain such lack of response resulting in sufficient protein synthesis to prevent atrophy.^38^ Moreover, the effects of repeated BoNT‐A treatment on muscle growth are controversial as after each injection muscles may atrophy and weaken which reduces their potential for adaptation.^5^, ^39^ However, studies in cerebral palsy have been inconclusive on the long‐term effects of BoNT‐A on muscle volume.^40^, ^41^ As muscles in children with SP are less developed (i.e., in this case ≈30% less volume of GM compared with that of typically developing children), it could also be that they may not be sensitive to atrophy any further. This would imply that muscle fibers had already reached the smallest possible cross‐sectional area and could not atrophy any further without losing fibers.^42^, ^43^

In this study, we used clinically applicable 3D ultrasound assessments to quantify the morphological characteristics of the GM.^12^ The accuracy of this assessment has been tested, showing that ultrasound muscle volume estimates are on average off by 3% and fascicle lengths by 6%, compared with immersed cadaver muscles.^12^ BoNT‐A injection in quadriceps muscles of rabbits induced a reduction in muscle mass of 31%‐50% after 1 month.^39^ The above indicates that our 3D assessments are sufficiently sensitive to assess the changes in morphology in response to BoNT‐A injection after 9 and 26 wk.

### 5.4 Large treatment effects on foot deformation

Post‐treatment, the orientation of the line connecting the insertion of the GM at the calcaneus with the center of the bimalleolar axis had changed into a more plantar grade angle with respect to the rest of the foot. This likely allowed the entire foot to move into dorsiflexion angles at relatively shorter triceps surae lengths. In addition, effects of foot flexibility were increased at 9 wk, but returned to baseline at 26 wk. Flexibility of the foot allows large movements within the foot without changes in triceps surae length. Returning to the mechanisms by which gait improved post‐treatment, we suggest, that in this case, the combination of BoNT‐A and serial casting likely reduced the rigidity of the foot to better cope with the AFO. It is concluded that the intervention and follow‐up had varying effects on the foot both at rest and when under load (i.e., between 0 and 4 Nm).

### 5.5 Limitations of the study

This report has some obvious limitations. Firstly, we report the observed results of one case study. To the best of the author's knowledge, this is the first comprehensive study reporting effects of BoNT‐A and serial casting in a subject with SP. Obviously, a case study is inherently not a generalizable research study, yet that was not the primary aim of the paper. Rather, we demonstrate what can be learned by instrumented muscle‐ and joint‐level assessments following a very commonly applied treatment.

A second limitation is the lack of functional assessment at the level of activities. We carried out assessments only on the “body functions and structures” of The International Classification of Functioning, Disability and Health.^44^

### 5.6 Clinical perspective and applicability

There is growing awareness among clinicians that subjective clinical examination of impairments is limited in terms of reliability, validity, and sensitivity.^10^, ^45^ Given concern of repeated use of BoNT‐A in growing muscle,^5^ maximum effort should be spent in developing more informative and robust assessment methods. Here, we present instrumented assessments that have been validated for clinical use in children with cerebral palsy.^11^, ^12^, ^13^, ^19^, ^46^ In this case study, we demonstrated that such in‐depth evaluation provided insight into the working mechanisms of treatment with BoNT‐A and serial casting. This in‐depth understanding of how changes in gait were achieved can be translated to better‐informed clinical decision‐making and individualized patient management. In our case, we found limited muscular morphological adaptation post‐treatment to explain the short‐term improvements in gait. Possibly, treatment resulted in a more flexible foot that could be fitted more easily (pain‐free) into an AFO. This result is similar to that of recent studies showing limited long‐term effects of stretching interventions, with increases in joint ranges being accounted for by increased tolerance.^30^, ^47^ While the child was better able to tolerate her AFO in the short term, it is questionable whether increasing foot flexibility is desirable and therefore whether treatment with serial casting following BoNT‐A was the best long‐term solution. In our patient's case, stretch reflex hyperactivity, rather than passive muscle properties, was very likely a strong determinant of her gait deviations. This was evident before treatment by a high amount of velocity‐dependent activation during passive stretch as well as by the quick return of this hyperactivity 26 wk post‐treatment with a subsequent return of the ankle dorsiflexion impairment. Therefore, in this case, a better long‐term solution that specifically targets the hyperactivity (rather than the passive muscle properties) may be selective dorsal rhizotomy. In other cases where hyperactivity is limited, but muscle properties are impaired, alternative treatment choices may be superior.

The above reasoning requires further validation by means of clinical research. Firstly, reference databases of typical as well as pathology‐specific spasticity, muscle morphology, and foot flexibility impairments as assessed with instrumented methods are essential. These will allow for context‐specific interpretation of any values obtained from individual patients. Secondly, investigations with large subject samples should be initiated to study the effects of commonly applied treatments on the impairments as assessed in an instrumented way. By doing so, we can better understand the treatments working mechanisms and start to tailor them in a muscle and patient‐specific way. Finally, given the lack of effective treatments to positively alter muscle properties in SP, more research in this field is urgently required.

## 6 CONCLUSION

Here, we show that BoNT‐A injections combined with serial casting and physiotherapy resulted in positive short‐term effects on gait, spasticity, and foot sole rotation. However, increased ankle joint ROM was largely explained by increased foot flexibility, rather than by changes in GM morphology, at which the treatment was targeted. After 26 wk, increased foot flexibility was not retained, while also spasticity and dorsiflexion joint restrictions returned to baseline values. The outcome of this study questions the rationale of administering BoNT‐A and casting to treat ankle dorsiflexion gait deviations in this case. Comprehensive assessments on multiple levels from muscle to joint to foot, helped establish the mechanisms underlying ankle dorsiflexion impairment and obtain insight in changes following treatment. Such a combination of assessments can provide valuable information for patient‐specific clinical decisions.

## CONFLICT OF INTEREST

None Declared.

## AUTHOR CONTRIBUTIONS

GW: conceived and designed the experiments, executed the experiments, analyzed and interpreted the findings, and drafted and revised the article. LS: conceived and designed the experiments, analyzed and interpreted the findings, and drafted and revised the article. LO: executed the experiments, and contributed to the analysis and interpretation of the findings, and draft and revision of the article. RTJ: contributed to the design of the experiments, the analysis and interpretation of the findings, and draft and revision of the article. JH: judged the experiments and manuscript particularly on technical aspects, and contributed to the revision of the article. AB: judged the experiments and manuscript particularly on clinical aspects and contributed to the revision of the article. LB: conceived and designed the experiments, executed the measurements, analyzed and interpreted the findings, and drafted and revised the article.

## References

[1] Gage JR . The Identification and Treatment of Gait Problems in Cerebral Palsy, 2nd ed London, UK: Mac Keith Press; 2009.

[2] Ahnert‐Hilger G , Bigalke H . Molecular aspects of tetanus and botulinum neurotoxin poisoning. Prog Neurogibol. 1995;46(1):83‐96. 10.1016/0301-0082(95)00003-e 7568911

[3] Boyd RN , Pliatsios V , Starr R , Wolfe R , Graham HK . Biomechanical transformation of the gastroc‐soleus muscle with botulinum toxin A in children with cerebral palsy. Dev Med Child Neurol. 2000;42(1):32‐41. 10665973 10.1017/s0012162200000074

[4] Tustin K , Patel A . A critical evaluation of the updated evidence for casting for equinus deformity in children with cerebral palsy. Physiother Res Int. 2017;22(1):e1646. 10.1002/pri.1646 26351821

[5] Gough M , Fairhurst C , Shortland AP . Botulinum toxin and cerebral palsy: time for reflection? Dev Med Child Neurol. 2005;47(10):709‐712. 16174320 10.1017/S0012162205001453

[6] Bar‐On L , Van Campenhout A , Desloovere K , et al. Is an instrumented spasticity assessment an improvement over clinical spasticity scales in assessing and predicting the response to integrated Botulinum Toxin‐A treatment in children with Cerebral Palsy? Arch Phys Med Rehabil. 2013;95(060799):515‐523. 23994052 10.1016/j.apmr.2013.08.010

[7] Desloovere K , De Cat J , Molenaers G , et al. The effect of different physiotherapy interventions in post‐BTX‐A treatment of children with cerebral palsy. Eur J Paediatr Neurol. 2012;16(1):20‐28. 21945796 10.1016/j.ejpn.2011.08.009

[8] Wiart L , Darrah J , Kembhavi G . Stretching with children with cerebral palsy: what do we know and where are we going? Pediatr Phys Ther. 2008;20(2):173‐178. 18480717 10.1097/PEP.0b013e3181728a8c

[9] Gracies JM , Bayle N , Vinti M , et al. Five‐step clinical assessment in spastic paresis. Eur J Phys Rehabil Med. 2010;46(3):411‐421. 20927007

[10] Fleuren J , Voerman GE , Erren‐Wolters CV , et al. Stop using the Ashworth Scale for the assessment of spasticity. J Neurol Neurosurg Psychiatry. 2010;81(1):46‐52. 19770162 10.1136/jnnp.2009.177071

[11] Sloot LH , van der Krogt MM , de Gooijer‐van de Groep KL , et al. The validity and reliability of modelled neural and tissue properties of the ankle muscles in children with cerebral palsy. Gait Posture. 2015;42(1):7‐15. 25936760 10.1016/j.gaitpost.2015.04.006

[12] Weide G , van der Zwaard S , Huijing PA , Jaspers RT , Harlaar J . 3D Ultrasound imaging: fast and cost‐effective morphometry of musculoskeletal tissue. J Vis Exp. 2017;(129):e55943. 10.3791/55943 PMC5755508 29286445

[13] Bar‐On L , Aertbeliën E , Wambacq H , et al. A clinical measurement to quantify spasticity in children with cerebral palsy by integration of multidimensional signals. Gait Posture. 2013;38(1):141‐147. 23218728 10.1016/j.gaitpost.2012.11.003

[14] Becher J , Doorenbosch C , Folmer K , Scholtes V , Voorman J , Wolterbeek N . Standaard Lichamelijk Onderzoek Bij Kinderen Met Een Centraal Motorische Parese. Amsterdam, the Netherlands: Reed Business bv; 2011.

[15] Huijing PA , Bénard MR , Harlaar J , Jaspers RT , Becher JG . Movement within foot and ankle joint in children with spastic cerebral palsy: a 3‐dimensional ultrasound analysis of medial gastrocnemius length with correction for effects of foot deformation. BMC Musculoskelet Disord. 2013;14(1):365. 24364826 10.1186/1471-2474-14-365 PMC3909357

[16] Grunt S , van Kampen PJ , van der Krogt MM , Brehm M‐A , Doorenbosch C , Becher JG . Reproducibility and validity of video screen measurements of gait in children with spastic cerebral palsy. Gait Posture. 2010;31(4):489‐494. 20304653 10.1016/j.gaitpost.2010.02.006

[17] Hof AL . Scaling gait data to body size. Gait Posture. 1996;4(3):222‐223.

[18] Sloot LH , Bar‐On L , van der Krogt MM , et al. Motorized versus manual instrumented spasticity assessment in children with cerebral palsy. Dev Med Child Neurol. 2016;59(2):145‐151. 27363603 10.1111/dmcn.13194

[19] Bénard MR , Jaspers RT , Huijing PA , Becher JG , Harlaar J . Reproducibility of hand‐held ankle dynamometry to measure altered ankle moment‐angle characteristics in children with spastic cerebral palsy. Clin Biomech. 2010;25(8):802‐808. 10.1016/j.clinbiomech.2010.04.010 20541856

[20] Hermens HJ , Disselhorst‐Klug C , Rau G . The recommendations for sensors and sensor placement procedures for surface electromyography In: Hermens H, Freriks B, Merletti R, et al. eds. SENIAM 8; European Recommendations for Surface Electromyography. Roessingh Research and Development B.V. In Seniam; 1999:13‐54.

[21] Tardieu C , Bret MD , Colbeau‐Justin P , Huet de la Tour E . Relationship of triceps surae torques to photographed tibia‐calcaneum angles in man (II). Eur J Appl Physiol Occup Physiol. 1977;37(2):153‐161. 902656 10.1007/BF00421701

[22] Nieuwenhuys A , Papageorgiou E , Pataky T , De Laet T , Molenaers G , Desloovere K . Literature review and comparison of two statistical methods to evaluate the effect of botulinum toxin treatment on gait in children with cerebral palsy. PLoS ONE. 2016;11(3):e0152697. 27030973 10.1371/journal.pone.0152697 PMC4816309

[23] Desloovere K , Molenaers G , Jonkers I , et al. A randomized study of combined botulinum toxin type A and casting in the ambulant child with cerebral palsy using objective outcome measures. Eur J Neurol. 2001;8(Suppl 5):75‐87. 11851736 10.1046/j.1468-1331.2001.00040.x

[24] Ackman JD , Russman BS , Thomas SS , et al. Comparing botulinum toxin A with casting for treatment of dynamic equinus in children with cerebral palsy. Dev Med Child Neurol. 2005;47(9):620‐627. 16138670

[25] Dunne JW , Singer BJ , Silbert PL , Singer KP . Prolonged vastus lateralis denervation after botulinum toxin type A injection. Mov Disord. 2010;25(3):397‐401. 20108381 10.1002/mds.22852

[26] Scholtes VA , Dallmeijer AJ , Knol DL , et al. Effect of multilevel botulinum toxin a and comprehensive rehabilitation on gait in cerebral palsy. Pediatr Neurol. 2007;36(1):30‐39. 17162194 10.1016/j.pediatrneurol.2006.09.010

[27] Wren T , Patrick Do K , Kay RM . Gastrocnemius and soleus lengths in cerebral palsy equinus gait—differences between children with and without static contracture and effects of gastrocnemius recession. J Biomech. 2004;37(9):1321‐1327. 15275839 10.1016/j.jbiomech.2003.12.035

[28] Tedroff K , Granath F , Forssberg H , Haglund‐Akerlind Y . Long‐term effects of botulinum toxin A in children with cerebral palsy. Dev Med Child Neurol. 2009;51(2):120‐127. 19191845 10.1111/j.1469-8749.2008.03189.x

[29] Pothrat C , Authier G , Viehweger E , Berton E , Rao G . One‐ and multi‐segment foot models lead to opposite results on ankle joint kinematics during gait: implications for clinical assessment. Clin Biomech (Bristol, Avon). 2015;30(5):493‐499. 10.1016/j.clinbiomech.2015.03.004 25812728

[30] Kalkman BM , Bar‐On L , Cenni F , et al. Medial gastrocnemius muscle stiffness cannot explain the increased ankle joint range of motion following passive stretching in children with cerebral palsy. Exp Physiol. 2018;103(3):350‐357. 29280208 10.1113/EP086738

[31] Love SC , Valentine JP , Blair EM , Price CJ , Cole JH , Chauvel PJ . The effect of botulinum toxin type A on the functional ability of the child with spastic hemiplegia a randomized controlled trial. Eur J Neurol. 2001;8(Suppl 5):50‐58. 11851734 10.1046/j.1468-1331.2001.00038.x

[32] Juzans P , Comella JX , Molgo J , Faille L , Angaut‐Petit D . Nerve terminal sprouting in botulinum type‐A treated mouse levator auris longus muscle. Neuromuscul Disord. 1996;6(3):177‐185. 8784806 10.1016/0960-8966(96)00041-7

[33] Cosgrove AP , Corry IS , Graham HK . Botulinum toxin in the management of the lower limb in cerebral palsy. Dev Med Child Neurol. 2008;36(5):386‐396. 10.1111/j.1469-8749.1994.tb11864.x 8168657

[34] Park ES , Sim E , Rha D‐W , Jung S . Architectural changes of the gastrocnemius muscle after Botulinum toxin type A injection in children with cerebral palsy. Yonsei Med J. 2014;55(5):1406. 25048504 10.3349/ymj.2014.55.5.1406 PMC4108831

[35] Schroeder AS , Ertl‐Wagner B , Britsch S , et al. Muscle biopsy substantiates long‐term MRI alterations one year after a single dose of botulinum toxin injected into the lateral gastrocnemius muscle of healthy volunteers. Mov Disord. 2009;24(10):1494‐1503. 19489066 10.1002/mds.22661

[36] Legerlotz K , Matthews KG , Christopher MD , Smith HK . Botulinum toxin‐induced paralysis leads to slower myosin heavy chain isoform composition and reduced titin content in juvenile rat gastrocnemius muscle. Muscle Nerve. 2009;39(4):472‐479. 19260067 10.1002/mus.21247

[37] Wang K , McCarter R , Wright J , Beverly J , Ramirez‐Mitchell R . Regulation of skeletal muscle stiffness and elasticity by titin isoforms: a test of the segmental extension model of resting tension. Proc Natl Acad Sci U S A. 1991;88(16):7101‐7105. 1714586 10.1073/pnas.88.16.7101 PMC52241

[38] Goldspink G . Changes in muscle mass and phenotype and the expression of autocrine and systemic growth factors by muscle in response to stretch and overload. J Anat. 1999;194(3):323‐334. 10386770 10.1046/j.1469-7580.1999.19430323.x PMC1467932

[39] Fortuna R , Vaz MA , Youssef AR , Longino D , Herzog W . Changes in contractile properties of muscles receiving repeat injections of botulinum toxin (Botox). J Biomech. 2011;44(1):39‐44. 20828699 10.1016/j.jbiomech.2010.08.020

[40] Barber L , Hastings‐Ison T , Baker R , Kerr Graham H , Barrett R , Lichtwark G . The effects of botulinum toxin injection frequency on calf muscle growth in young children with spastic cerebral palsy: a 12‐month prospective study. J Child Orthop. 2013;7(5):425‐433. 24432106 10.1007/s11832-013-0503-x PMC3838523

[41] Schless S‐H , Cenni F , Bar‐On L , et al. Medial gastrocnemius volume and echo‐intensity after botulinumneurotoxin A interventions in children with spastic cerebral palsy. Dev Med Child Neurol. 2018. [Epub ahead of print]. 10.1111/dmcn.14056 30320442

[42] van der Meer S , Jaspers RT , Jones DA , Degens H . Time‐course of changes in the myonuclear domain during denervation in young‐adult and old rat gastrocnemius muscle. Muscle Nerve. 2011;43(2):212‐222. 21254086 10.1002/mus.21822

[43] Sartori R , Schirwis E , Blaauw B , et al. BMP signaling controls muscle mass. Nat Genet. 2013;45(11):1309‐1318. 24076600 10.1038/ng.2772

[44] World Health Organization . International Classification of Functioning, Disability, and Health: Children & Youth Version: ICF‐Y Geneva: WHO; 2007.

[45] van den Noort JC , Bar‐On L , Aertbeliën E , et al. European consensus on the concepts and measurement of the pathophysiological neuromuscular responses to passive muscle stretch. Eur J Neurol. 2017;24(7):981‐e38. 28557247 10.1111/ene.13322

[46] Cenni F , Monari D , Desloovere K , Aertbeliën E , Schless S‐H , Bruyninckx H . The reliability and validity of a clinical 3D freehand ultrasound system. Comput Methods Programs Biomed. 2016;136:179‐187. 27686714 10.1016/j.cmpb.2016.09.001

[47] Hösl M , Böhm H , Arampatzis A , Döderlein L . Effects of ankle-foot braces on medial gastrocnemius morphometrics and gait in children with cerebral palsy. J Child Orthop. 2015; 9(3):209-219. 10.1007/s11832-015-0664-x PMC4486505 26108740

